# An Online Database of the Immatures of Coleoptera (Arthopoda, Insecta) Described from Brazil

**DOI:** 10.3897/BDJ.5.e12252

**Published:** 2017-04-06

**Authors:** Vinicius S. Ferreira, Cleide Costa

**Affiliations:** 1 Montana State University, Bozeman, United States of America; 2 Museu de Zoologia da Universidade de São Paulo, São Paulo, Brazil

**Keywords:** Neotropical Region, Larvae, Pupae, South America, Beetles.

## Abstract

**Background:**

An online database of the described immature beetles from Brazil is presented for the first time based on published literature. The main purpose of this online database is to ensure accessibility to data associated with the described immature Coleoptera from Brazil, which will be useful for future biological, ecological, conservational and biogeographical studies.

**New information:**

More than 9,486 specimens of 248 genera, 282 species and 4 subspecies of 76 Coleoptera families from 15 states and the Federal District of Brazil were found. Taxonomical and ecological information about each species, when available, are given. The dataset of Immatures of Coleoptera described from Brazil are available and can be accessed through the portals of GBIF at http://www.gbif.org/dataset/8e0e9330-e1b2-475a-9891-4fa8e5c6f57f and the SiBBr at http://ipt.sibbr.gov.br/sibbr/resource?r=coleoptera_immature_of_brazil.

## Introduction

Chavan and Penev (2011) stated that “*one of the effective strategies for addressing the growing biodiversity crisis is access to a range of biodiversity- and ecosystems-related data and information in a useful form to fill the extensive biodiversity knowledge gap that exists today*”. The authors proposed “*the biodiversity data paper as a mechanism to incentivize efforts and investment towards discovery and publishing of biodiversity data resources*”.

*Following their definition, a data paper is a journal publication whose primary purpose is to describe data, rather than to report a research investigation* (*Chavan and Penev 2011). As such, a data paper will contain facts about data but no hypotheses or* arguments in support of hypotheses and theories based on data (Chavan and Penev 2011). Still according to these authors, the purposes of a data paper can be summarized in three: (1) provide a citable journal publication that brings credit to the data publisher, (2) describe the data in a structured and human-readable format and (3) to bring the existence of that data to the attention of the scientific community, and these three points are exactly what we wanted to do about the knowledge of immature beetles in Brazil.

The knowledge of the immature beetle’s diversity is still very incipient and the task of the Coleopterists to identify and determine beetles at this stages is enormous. Larvae and adults of Coleoptera possess a mix of dependent and independent adaptations that reflect a complex evolution. Taxonomic and phylogenetic analyses that involves characters of larvae and adults could offer a wider vision of the evolutionary process.

Besides the taxonomic and systematic perspective, the study of the whole life cycle of Coleoptera is very important for the economic, environment and medical perspectives, especially because many immatures of beetles are responsible for considerable financial losses in crops, trees and other aspects that impact on peoples lives.

In this paper, an online dataset of the immatures of Coleoptera described from Brazil is provided for the first time. In this dataset, for each species, information about the amount of specimens studied, development time, localities, host plants and other relevant information is given.

## Sampling methods

### Sampling description

The bibliographic survey was made by means of The Zoological Record Online version which is an electronic index of Zoological literature. The terms used in the online searches were several combinations of the following key words: Coleoptera, Immature, Imaturos, Immatures, Larvae, Larva, Pupa, Pupae, Brazil, Brasil, Beetles, Besouros.

The time span of the online search is from 1864 to the current year of 2017 (February) and a total of 127 publications were used to compose this dataset. We have also consulted the literature published by Costa and collaborators, which include 27 scientific articles dealing with the specimens of the MZUSP collection in a series entitled “Larvae of Neotropical Coleoptera” and a book on the “*Larvas de Coleoptera do Brasil*”, in 1988 (Costa et al. 1988). Although numerous immature descriptions in many publications were made based on correlation with adults in field or posterior association most of the specimens of Costa and collaborators were reared in laboratory under controlled conditions, thus ensuring a high level of certainty in the identification of immature beetles.

The majority of specimens described in the literature and enrolled in this datapaper (about 8,131) belong to the Collection of the “Museu de Zoologia da Universidade de São Paulo” (CIC-MZUSP). This collection is maintained in glass flasks of several sizes, with about 51,000 specimens maintained in 70% ethanol (Costa 2010, Barbosa et al. 2013). Most of the adults are stored together with the immature reared in the laboratory, and part of the adults are mounted and kept in separate cabinets associated with the immature collection.

## Geographic coverage

### Description

The sampling area of this work is delimited by the current administrative boundaries of the Brazilian territory. Many species are from the Atlantic Forest and “Cerrado” vegetation in Central Brazil, part of the Amazon Forest and the Restinga areas in the South Coast of São Paulo and Rio Grande do Sul states. We found specimens of immatures of Coleoptera described from 15 states and the Federal District and 137 counties.

As most of the papers used to compose this dataset did not present the exactly geographical coordinates of the localities, the geo referencing of the localities was made using the geoLoc tool <http://splink.cria.org.br/geoloc>, a tool developed by CRIA to assist biological collections in geo referencing their data. Their database includes approximately 750,000 of Brazilian names localities.

## Taxonomic coverage

### Description

According to Bouchard et al. (2011) about 125 families of Coleoptera occurs in Brazil and just a tiny number of immatures are known. This dataset corresponds to more than 9,486 specimens of 248 genera, 282 species and 4 subspecies of 76 families (which included eggs, larvae, pupae, pre-pupae and their exuviae) of immatures of Coleoptera that were described from Brazil. The identifications and classification of the taxa were made by the authors of each publication. We claim no responsibility for any potential misidentification in the data provided in this dataset.

## Traits coverage

From the literature consulted the specimens were collected in many habitats, that are summarized below. The precise information on each specimen, when provided, is on the dataset.

- Specimens were originally found in wooden floors inside residences, under decaying wood of different kinds of trees, in pasture fields, inside holes in the grass or inside galleries of dead trunks and bounds, in litter and on beach and at night walking in arenous railroad.
- In aquatic environments specimens were found in streams of water close to rocks, in aquatic rocky wall, temporary pounds and lagoons.
- Associated with other Insects, Arthropods and mammals and birds and their excrements the specimens were found in subterranean nest of *Geotrigona* sp. (Apidae, Meliponinae), in dead bodies of rats, chicken and hedgehog; in epigeum nests Isoptera of *Anoplotermes*, *Microcerotermes* sp., *Nasutitermes* and *Cornitermes* sp., in excrements of bird, equines, bovines and swine, preying on Homoptera, Psyllidae and inside Mantidae ootheca.
- Associated with vegetables and fungi immatures were found in hard trunks of Lauraceae, probably of *Nectranda* or *Ocotea*, in bromeliads with vegetal detritus, in the axil of leaves, sugar cane plantation, in *Casuarina* sp. and in dried cut bamboos as well as inside many fruits and leaves of several families of plants and associated with fungi.

## Usage rights

### Use license

Other

### IP rights notes

Creative Commons - Attribution 4.0 International (CC BY 4.0)

## Data resources

### Data package title

An Online Database of the Immatures of Coleoptera (Arthopoda, Insecta) Described from Brazil

### Resource link

http://www.gbif.org/dataset/8e0e9330-e1b2-475a-9891-4fa8e5c6f57f

### Alternative identifiers

http://ipt.sibbr.gov.br/sibbr/resource?r=coleoptera_immature_of_brazil

### Number of data sets

1

### Data set 1.

#### Data set name

An Online Database of the Immatures of Coleoptera (Arthopoda, Insecta) Described from Brazil

#### Data format

Darwin Core

#### Number of columns

1

#### Character set

UTF-8

#### Data format version

http://rs.tdwg.org/dwc/terms/

#### 

**Data set 1.** 

Column label	Column description
http://rs.tdwg.org/dwc/terms/	See terms in the link.
